# Hospital and Institutional News

**Published:** 1912-03-09

**Authors:** 


### HOSPITAL AND INSTITUTIONAL NEWS.

### THE ADVISORY COMMITTEE ON NATIONAL INSURANCE.

At the invitation of the National Health Insurance Committee the British Hospitals Association have submitted, for a selection to be made, four names of members willing to serve on the Advisory Committee. They are Mr. A. William West (St. George's Hospital), Mr. E. Stewart Johnson (Hospital for Sick Children), Dr. D. J. Mackintosh (Glasgow Western Infirmary), and Mr. H. Wade Deacon (Chairman Liverpool Royal Infirmary).

### A ROYAL VISIT TO THE WEST LONDON HOSPITAL.

With very brief warning the West London Hospital received a visit from the Queen last Saturday. Owing to his absence in Scotland, the Duke of Abercorn, chairman of the hospital, was unable to be present, but in his absence her Majesty was received by Mr. H. A. Madge, the financial secretary, Mr. A. Betteridge, the secretary, while the house committee was represented by Mr. Talbot Rice. On behalf of the medical staff, Dr. Seymour Taylor, senior physician, and Mr. Swinford Edwards, senior surgeon, were also present. Her Majesty, who brought several baskets of flowers, stayed about an hour in the building, and talked to several of the patients, according to her gracious custom when making these visits.

### THE SOUTH MANCHESTER ELECTION.

The result of the South Manchester election, by which the Ministerial candidate, Sir A. Haworth, has been defeated, will come as a relief to the commonsense members of the public. It proves once more that in England party interests will never be permitted to override the principles of justice and fair dealing. The result is largely due, no doubt, to the general feeling throughout the country on this measure that, the medical profession and the voluntary hospitals have been treated unjustly to the detriment of the public interests. Voluntary hospital managers, therefore, may see in this election an importance, which can hardly be exaggerated. It is is the most powerful blow which has yet been struck against the Insurance Act as it stands, and is consequently not merely a victory for the medical profession, but the first step towards an amending Act which must contain provisions to secure hospital benefits for the insured. Whatever view the party press may take upon this election its significance cannot be missed by the Government or the Opposition. The evidence this election affords of the practical influence which medical and hospital questions may have upon the electorate, irrespective of party, cannot longer be disregarded by any political leader. The error into which the politicians have fallen may be attributed to the fact, that, neither the medical profession nor the interests of the voluntary hospitals are at present adequately represented in the House of Commons.

### WOMEN SUFFRAGISTS AND HOSPITAL WINDOWS.

The campaign of violence which the societies anxious to secure votes for ladies have recommenced, has not spared the hospitals. Mr. John Henry Johnson, secretary of the Royal Westminster Ophthalmic Hospital, complains that on Monday night two windows were broken which were of special manufacture and not insured. We cannot do better than quote his own comment—namely, that current expenses are sufficiently difficult to meet, without these women, whose tactics have increased the number of hospital patients before now, adding to the extraordinary expenditure out of sheer wantonness.

### A HERO OF HOSPITAL CONTROVERSIES.

The early death of Mr. J. Troutbeck, the coroner for the South-West district of London, removes from amongst us one with whom hospital medical officers had many and bitter controversies. Of recent years the ill-feeling that once existed has died down a good deal, though it would be idle to pretend that the profession viewed with approval everything that this coroner did. The last occasion upon which there was any really strong resentment was when Sir Victor Horsley was subpœnaed to an inquest held upon a patient who died after some severe operation. From the fact that the procedure was not repeated, it may be inferred that Mr. Troutbeck saw the unwisdom of it. It gives us pleasure now to express entire confidence in Mr. Troutbeck's honesty and singleness of purpose, both in this particular case and in the bickerings of previous years. We feel sure that he was quite wrong over several points, but we also feel that he was quite sure he was doing right. The moral of the whole story is that every coroner ought to be a medical man as well as a lawyer. For Mr. Troutbeck's capacity in matters legal we always held the very highest respect. Apart from his peculiar views upon certain medical matters, he was a man whose gifts enabled him to perform his duties in the most wholly admirable manner. We may add that to medical witnesses individually he always showed the greatest courtesy and consideration.

### HOSPITAL EMPLOYEES UNDER THE INSURANCE ACT.

A representative gathering of the medical and nursing professions of Scotland, with Lord Inverclyde in the chair as President of the Association for Promoting the Registration of Nurses in Scotland, was held at Edinburgh on the 27th ult. It was reported that no progress had been made during the year in regard to the registration of nurses, because National Insurance had occupied the whole time of Parliament. Dr. Mackintosh, the hon. secretary, stated that the membership now amounted to 2,270, of whom 179 had joined during 1911. He invited Mr. Dick, the secretary of the Royal National Pension Fund for Nurses, to attend and explain fully in what respect the National

Insurance Act affected nurses, and how they could adequately protect their own interests. Mr. Dick explained that the separate section of the Pension Fund would be open to all women nurses, whether members of the Pension Fund or not. He was glad to have the opportunity of explaining the position of nurses under the National Insurance Act, and of giving reasons why it was desirable they should join the special branch of the Pension Fund. The separate section would not be carried on for profit, and its affairs would provide that it should be subject to the absolute control of its members. Some interesting details of the position of the employees of voluntary hospitals under the National Insurance Act will be found on another page of the present issue.

### THE ADMIRALTY YACHT AS A HOSPITAL.

In answer to a question with reference to the Admiralty yacht, in the House of Commons, Mr. Churchill is reported to have said that the Admiralty yacht becomes a hospital-ship in time of war. It is interesting to know that the Admiralty yacht has the capacity for this very important change, which, as anyone who has inspected hospital-ships knows, is a highly complicated and difficult matter. The confidence of hospital workers in the yacht's adaptability will perhaps not be increased by Sir C. Kinloch-Cooke's assertion that in time of peace the yacht "mainly serves the purposes of a hotel." In view of the proposed hospital-ship for the Navy, we can only hope that a strictly technical standard will be set up for such vessels in the future.

### A LADY PATRON'S EXAMPLE.

Among those persons whose help to the London hospitals of recent years deserves special mention the Duchess of Marlborough stands almost alone. It would be difficult to find another patron who took a more generous view of his or her duties. Apart from opening many functions in connection with the West Ham and Eastern General Hospital, she recently gave an open-air balcony to the children's ward, and since then has doubled her annual subscription. It is also interesting to note that the experimental appointment of a lady almoner has been made a permanency, Miss Gregory having given satisfaction at the end of her first six months of work. Mr. T. A. Cook, the chairman, has taken our account of the magnificent achievement of the Leicester working-men to heart, and recently reminded an audience at West Ham that that district too was an industrial one, and that the recent increase of income was not due to local support. The new members of the committee, who we hope will impress this view in the locality wherever they go during the present year, are Mr. R. Bennett, Mr. James Copper, and Mr. S. Fredericks. The chaplain, who no doubt has all his work cut out in visiting patients and maintaining the claims of Hospital Sunday, is the Rev. F. J. Key.

### STATE AID AND TROPICAL RESEARCH.

Mr. Lewis Harcourt at the Mansion House last week made an interesting speech on behalf of the London School of Tropical Medicine, in which, while pleading for £20,000 to form an endowment fund, he summarised the Government's assistance of tropical research. He declared that £50,000 had been spent by the Treasury on the investigation of sleeping-sickness in Uganda, and £8,000 was being spent every year on entomological and tropical research and on Sir David Bruce's expedition to Nyasaland for the study of sleeping-sickness. The senior teacher and physician to the school, Sir Patrick Manson, remarked that a man now leaving for tropical Africa need not feel that he was going to his grave. We are happy to add that the sum of £2,393 was announced as the result of the meeting. There is no doubt that this year the Mansion House does double service to the cause of science and hospitals, through the presence of Sir Thomas Crosby, who, as Lord Mayor, can represent these interests in a way that has been impossible for his predecessors.

### FURTHER DISTINCTIONS FOR MEDICAL MEN.

In the latest list of promotions in and appointments to the Royal Victorian Order occurs the name of Mr. William Joseph Essery, M.B., M.R.C.S., who has been appointed a Commander. Mr. Essery has held appointments at the Royal Portsmouth Hospital and at the Salop Infirmary. Mr. John Astley Bloxam, F.R.C.S., Surgeon-General Hayward Reader Whitehead, C.B., F.R.C.S., and Dr. William Edmund Cant, M.D., have been promoted Knights of Grace in the Order of the Hospital of St. John of Jerusalem in England. Mr. Bloxam and Dr. Cant have previously been Honorary Associates.

### WAKE UP THE ANNUAL MEETING.

Mr. Godfrey Hamilton, the Secretary of the National Hospital for the Paralysed, exhibits great taste and some originality in the literature which he produces and circulates amongst the public. His latest venture is the circulation of a notice of the annual general meeting, accompanied by a white card containing a green notice, entitled "Extracts from the Press." This intimates to the Governors the illustrious persons who are to attend the annual meeting, and should attract a large attendance to Devonshire House on the 29th inst. A further attraction to the recipients is a little old-fashioned-looking booklet in blue boards, containing some facts about the hospital and its work, with illustrations. Taken together, few simpler or more attractive aids to liberal giving have been issued on behalf of a voluntary hospital. Mr. Hamilton is to be congratulated because we are convinced that too little use is made of the annual meeting as a means to increase the supporters and revenues of the voluntary hospitals.

### THE FRUITS OF ADVICE AT COLCHESTER.

Some time ago The Hospital put forward a series of suggestions for helping the Essex County Hospital out of the financial difficulty in which what looked very like the indifference of Colchester had placed that institution. The past year's accounts examined in the light of these suggestions have a certain interest. In the first place expenditure has exceeded income by some £1,200, in spite of the fact that the income is said to have improved by over £1,000. This increased income has largely been due to the efforts of the Workers' Committee, which in its first year has raised £345, and to the Ladies' Association, which produced £188, together with the gifts of the Ladies' Linen League, which Mrs. Egerton Green has inspired from the first. It may readily be stated, therefore, that where our suggestions have been followed the income has been increased as we foretold. The one suggestion which has not been adopted is that for a Colchester Hospital Association, each member of which should be asked to raise at least 5s. a year. Were our advice followed on this point also, the fresh deficiency, due apparently to a further increase of patients, might also be provided for. Mr. W. W. Hewitt, the president, for whose recent accident we are sincerely sorry, has set a fine example by raising his annual subscription to fifty guineas.

### DR. BOND'S SUCCESSOR AT LONG GROVE.

Dr. David Ogilvy, the senior medical officer at Horton Mental Hospital, has been appointed to succeed Dr. C. H. Bond, the new Lunacy Commissioner, as medical superintendent of Long Grove Mental Hospital. Dr. Ogilvy is an M.D. of Dublin, and his previous appointments include the posts of assistant medical officer at the West Riding Mental Hospital, Wakefield, and at Banstead Mental Hospital.

### A PROMINENT BIRMINGHAM SURGEON RESIGNS.

Sir Thomas Frederick Chavasse, F.R.C.S., senior surgeon, has resigned the post of honorary surgeon to the General Hospital, Birmingham. Allusion has been made to his great services for the past thirty years, and a sub-committee has been appointed to consider what steps should be taken to commemorate his services to the hospital. Sir Thomas Frederick Chavasse, who is an M.D. and F.R.C.S. Edinburgh (1878), was appointed assistant surgeon to the General Hospital in November 1878. He was appointed honorary surgeon as far back as December 1881.

### RADIUM AT THE CANCER AND MIDDLESEX HOSPITALS.

We understand that Sir David Salomons has lent, on certain conditions, twenty-five milligrammes of pure crystallised radium bromide for the treatment of cases at the Middlesex Hospital. In this connection it is interesting to note Dr. Robert Knox's remarks, as director of the new radio-therapeutic department of the Cancer Hospital, that "cases treated by radium might improve, but at present I see no indication that radium will replace operative measures in any particular case." The moral of this, from the hospital's point of view, is, of course, that any increase in the supply of radium at their disposal will be doubly valuable at our present early stage of knowledge, for the purposes of further experiment.

### THE NORWICH HOSPITAL AND THE EYE INFIRMARY.

As an alternative to the original plan of building special quarters in the grounds of the Norfolk and Norwich Hospital, a scheme based upon the report of a joint committee of the Eye Infirmary and the Norfolk and Norwich Hospital has been put forward. As Mr. R. E. Horsfall explained at a meeting presided over by Sir Peter Eade, under the present scheme the hospital will purchase the Grove House property, and lease it, at a nominal rent, to the Eye Infirmary, which will undertake repairs, build an out-patient department, and be managed by a committee of fifteen men, five of whom will be appointed by the hospital's board. The Eye Infirmary will also have the free services of one of the hospital's resident staff and will receive all ophthalmic cases sent by the hospital for treatment. All that need be said of this scheme at present is that it is the final outcome of a long discussion, in which unanimity has not been easy to arrive at.

### THE BRADFORD NEW INFIRMARY SCHEME.

Apparently the realisation of Mr. W. A. Pite's design for the proposed new infirmary at Bradford is to be postponed "until," in the words of the report, "a definite and practical policy has been formulated." To form this is surely the next business of the Infirmary's committee, now that the comprehensive scheme for a new hospital of 400 beds, at a cost of £200,000, has been modified to one of 260 beds at a cost of not more than £180,000. Mr. Jacob Moser criticised this total estimate, and recalled his opinion expressed three years ago that no more than £100,000, apart from the cost of the land, should be spent on a building with 300 beds. He was of opinion that an infirmary could be built at a cost of £350 per bed, whereas the estimate in the report of £180,000 for 260 beds worked out at about £692 per bed.

### THE NEW MEDICAL OFFICER FOR THE CITY.

Our readers will learn with interest that a proposal is to be made to amalgamate the post of Medical Officer for the City, which Dr. Collingridge resigned recently, with that of Medical Officer of Health for the Port of London. As Dr. Collingridge stated in his interview to our representative at the time when his resignation was announced, he himself had previously been Medical Officer to the Port. Dr. Herbert Williams at present holds that post, and though the work of the two offices has multiplied very much of late years, the above proposal is not altogether a surprise.

### A MODERN COMMITTEEMAN'S DUTIES.

Mr. Charles Hyde is to be congratulated upon the exceptionally interesting speech which he delivered at the Birmingham Children's Hospital. In speaking of the duties of hospital committeemen, Mr. Hyde emphasised one side of their work —a side that we have lately been impressing on the chaplains—which may be stated in his own words: "It is the duty of every member of the committee not only to attend the meetings and to take an active interest in the work of the hospital, but to talk about it on all convenient occasions, with a view of interesting all likely subscribers. In America the hospitals have special publicity committees, but this was unnecessary if every member of the committee acted as an advertising agent for the institution." If every hospital speaker were as vigorous as Mr. Hyde, hospital meetings would be more popular with the public than they are. Another interesting subject introduced by the speaker was the possibility of forming a co-operative committee for Birmingham, modelled upon the lines of King Edward's Hospital Fund. We should be glad to hear Mr. Hyde's views on the practical details of this important proposal, which he hopes has now passed beyond the stage of informal discussion.

### A COTTAGE HOSPITAL FOR HAMPTON.

Mr. W. A. Sherwood, chairman of the Hampton District Council, states that an anonymous gentleman has decided "to build, equip, and endow" a cottage hospital for Hampton. The plan, from these meagre details, seems comprehensive, and we shall look forward to giving further details at a later date.

### CONGRATULATIONS FOR A BRIGHTON SECRETARY.

The Brighton and Hove Lying-in Hospital has recently received the two most valued compliments which perhaps it is possible to attach to any institution. It has been complimented for its atmosphere, and complimented for its secretary. The Vicar of Brighton recently took the opportunity of delivering a speech on the value of this atmosphere, and of what has come to be called the hospital spirit in scientific institutions. He might have added that it is often to seek in the State hospitals of the Continent. The compliment paid to Mr. Leonard Holmes, the honorary secretary, was twofold. "He has," Mr. Ball Dodson recently remarked, "two extraordinary gifts not common to secretaries, perspicacity and perspicuity. He always gets to the heart of a thing, and is able to explain it in the simplest manner." Mr. Holmes, we understand, is somewhat anxious about the effect of the Insurance Act upon his institution. He foresees some modification of its district work, on the ground that if women are to receive a maternity benefit of thirty shillings it will be questionable how far a charitable institution should attend such cases. The question is further complicated by the fear of some antagonism on the part of local practitioners to some of the hospital's work, and by the difficulty of arranging what share the hospital and the medical men would be justified in taking of the thirty shillings. Whether in this connection or not, Admiral Bingham, chairman of the committee, has hinted at a new scheme, now under consideration, the details of which we shall hope to hear shortly.

### THE BOLTON COMPETITION.

The assessor, Mr. J. B. Gass, F.R.I.B.A., has now given his award in the competition, limited to architects practising in the borough, for a design of a nurses' home. Eleven designs were submitted, and Mr. Gass has awarded the first premium, £30, to Messrs. Henderson and Brown; the second, £20, to Messrs. Marshall Robinson, Son, and Wheeler; and the third, £10, to Messrs. Smith and Son. The estimated cost of the work is £8,700, and the new block is to be known as an "Edward" memorial.

### A COTTAGE HOSPITAL MORTUARY.

It must have been a great relief to Mr. K. Whetsone, secretary of the Southwold Cottage Hospital, to be able to announce that arrangements have been made with the town for a proper mortuary. His admission that previously "it had not been fit for anyone to place their dead in" no doubt is the truth, for all experience shows that otherwise entirely admirable cottage hospitals often are careless about their mortuaries, or else rely upon the local authorities altogether. Where the cottage hospital is very small, the number of patients scanty, and deaths consequently rare, there is something to be said for not expending money upon a virtually unused building. But once a cottage hospital adopts a mortuary at all, the same high standard must be demanded in this as in all other portions of the institution.

### SUCCESSFUL WORK AT A WESTON SANATORIUM.

The election of a new president to the Royal West of England Sanatorium at Weston, in the person of Mr. Herbert Henry Wills, J.P., draws attention to the present position of the institution. It is now free from debt, as Mr. W. Dealtry, the honorary secretary, shows in his report, and a special appeal for funds to supply a new sea-water settling tank and baths has been successful. This is a matter of some importance, since a principal feature of this sanatorium is its sea-water baths. A new rule has recently been passed for the non-admission of phthisical patients, but the public fails to realise this, as the large number of applications from such cases still shows. Mr. T. W. Warry is again honorary treasurer; and Colonel Whitting, chairman of the executive committee, has recently voiced the high appreciation in which the lady superintendent, Miss Mawe, is held. The sanatorium not being for phthisical cases, the uncertain outlook caused by the Insurance Act does not affect it to the same extent as other institutions.

### A WARD DEDICATED TO A SECRETARY.

The recent opening of the new St. Paul's Eye Hospital in Liverpool, in memory of the late founder, Mr. G. E. Walker, was noteworthy among other things from the fact that one of the wards has been dedicated to Mr. Crosbie Oates, the late honorary treasurer and secretary. Mr. Fred. Stubbs, chairman of the hospital committee, drew attention to this in his speech, and to the secretary's work. We think that the dedication of a ward to a secretary is a fact worth recording, not only because of the worker in whose memory it has been done, but because the instances are few in which the institutional staff is associated with the hospital in this graceful manner.

### FIFTY YEARS ON COMMITTEE.

It is not often that a speaker at a hospital meeting can illustrate his institution's progress by reminding his audience of what took place fifty years ago, when he was first elected a member of the committee. Mr. G. Soltau Symons was able to do this the other day and gave some interesting examples of the work of the South Devon and East Cornwall Hospital in 1862. Then, he said, subscriptions amounted only to £750. To-day the income, apart from legacies, is £8,136; but the recent installation of hot water and heating apparatus has led Sir Alfred Croft, K.C.I.E., the treasurer, to propose a sale of stock, in order to pay for this and for the enlargement of the theatre cost, the total amount being some £4,000.

### THE ALMONER IN THE MIDLANDS.

So successful has the experimental almoner proved during the past twelve months at the Birmingham General Hospital, that the committee has decided to go further and to appoint a permanent paid almoner. A lady almoner has been sent to London to be trained by the Almoners' Council; at the end of this period she will start regular work at the hospital. This is only the latest sign of the value of this new profession, the work of which seems more likely than perhaps any other institutional activity to grow in importance every year.

### THE MINERS AND NEWCASTLE INFIRMARY.

Thanks to legacies and to the support of the Northumberland and Durham miners, the Newcastle Royal Victoria Infirmary converted a deficit of £3,024 into a balance in hand of £856. It seems, however, that the number of cases received from the county of Durham are considerably more numerous that those from Northumberland, with the result that during the past six years there is a deficiency of £7,125 in the receipts for cases from Durham. We may note also the new proposals for regulating admissions, which Mr. W. Sutton, jun., has submitted. These would substitute one medical authority, the resident officer, for the various members of the honorary staff, who have at present the right of placing names on the waiting list. Sir William Stephenson, the Lord Mayor, is full of hopes for the continued financial prosperity of this hospital, which he bases upon "the better look-out all round of the trade of the county, but for the present coal dispute."

### TWO MORE REFERENCE WORKS.

The subject-matter of reference works is not, as a rule, amusing in itself, but the reverse is the case with *Truth* Cautionary List for 1912, a book which all up-to-date secretaries should appreciate. When inquiries have to be made—and for hospital secretaries this is a daily experience—a glance at this Cautionary List will often prove helpful. It is, in fact, the adjunct and supplement of all the other reference books. Of the latter, "Nisbet's Medical Directory" is a convenient volume, and its alphabetical list of practitioners provides the quickest way of tracing every qualified man, whether practising in town or country, north or south. A new and useful feature is the addition in full of all Christian names. At 8s. 6d. net it should be very popular.

### KING EDWARD MEMORIAL AT EASTBOURNE.

The complimentary dinner which has been announced to Mr. H. D. Farnell, Dr. T. Macqueen, and Dr. A. P. Sherwood, on their retirement from the staff of the Princess Alice Memorial Hospital, Eastbourne, is of interest as they have been connected with the hospital since its foundation almost twenty-nine years ago. Mr. Farnell actually is the last to retire from the original staff. Their retirement, moreover, marks an important date in the life of this hospital, since the new wing is being built, and the King Edward Memorial, of which this wing is to form part, is in the throes of accomplishment. This means, first, that the difficult task of raising money—for it has proved difficult in Eastbourne—has to be completed, and secondly, that the hospital itself will become a larger and more complex institution. As Mr. W. F. Wells, the honorary treasurer, recently stated, the hospital is growing into the premier charity of Eastbourne. The above-mentioned dinner should be used, together with its primary purpose, for pressing the hospital's claims upon the town, and certainly Mr. Farnell's parting words on a recent occasion—"the interests of the hospital are identical with the interests of the patient"—provide a capital text in this direction.

### AFTER-CARE IN BIRMINGHAM.

Another interesting example of after-care comes to hand from the report of the Birmingham Children's Hospital, which created a new department for this purpose a year ago. Mr. H. K. Beale, the chairman, has recently stated his belief in the value of this work, and has congratulated the ladies of Birmingham on following the example of the United States and Sweden in this matter. Miss Bartleet has presented a report in which the work is described, seventeen children having been taken in charge, and some of these not merely visited weekly, but provided with daily supplies of food and eggs from the hospital.

### MEDICAL OPPOSITION TO THE SCHOOL CLINIC.

Where medical opinion has not been the initiator of the school clinic, its promoters, as now at Ealing the Town Council, sometimes receive unexpected opposition from local practitioners. The medical men of Ealing, for instance, have objected to the post of medical officer being advertised, on the ground that no doctor must be appointed for more than twelve months; and indeed have forbidden any member of the profession to accept the post for a longer period. Another school clinic is to be opened at West Ham, but we are not aware that friction has occurred there on this question.

### AN INSTANCE OF HOSPITAL ADVERTISING.

A real tide of prosperity seems hardly too strong a phrase to describe the recent finances of the Royal National Orthopædic Hospital. Since 1909 the building debt has been reduced from £23,000 to about £5,000, and last year donations and annual subscriptions practically doubled. The

King's Fund and Sir Ernest Cassel deserve some of the praise, but for the secret of much of this success we must go, presumably, to Mr. Morley, the secretary. As the Duke of Marlborough remarked, it is now a question for the house committee to decide whether the 60 or 70 beds which were closed for want of money should be opened. A new scheme has been completed for the supply of surgical instruments to the hospital patients which is expected to hasten this work. The Earl of Denbigh, chairman of the committee, stated at the recent meeting that last year their appeals had been very successful, and "they had cast their net wide." This certainly seems an instance of successful advertising.

### MEDICAL TEACHING FOR COLONIAL CHILDREN.

A new volume is Miss Bower's *Simple Talks on Health* (Macmillan, 4d.), which also contains nothing but the truth, though it is too small to encompass the whole truth. This unpretentious pamphlet is rather too technical for an uneducated man or woman, and hardly full enough for an intelligent person of the more cultivated class. From South Africa an excellent sixpenny pamphlet reaches us, written by Mrs. R. C. Dauglish, entitled *The Anopheline Mosquito* (John Murray). This is addressed specifically to the children of the Empire, and it explains in language suited to a child's understanding the essence of the malaria problem and of the fight against mosquitoes. As far as the British Isles are concerned, our young people have no need of Mrs. Dauglish's help; but in nearly all our colonies the exact reverse is true. This idea of explaining to juvenile audiences the reason for anti-malarial measures is a most excellent one. It was written, we are told, for use in schools in the Barberton district of the Transvaal, one of the really bad malarious districts of that colony, as the Hampshire Regiment discovered in the war. May the day soon come when instruction such as this booklet provides will be given in every school in our tropical and sub-tropical colonies!

### A BAD PRACTICE OF UNQUALIFIED PHARMACISTS.

It is disconcerting to observe that a practice has grown up among unqualified persons of selling certain standard preparations—which should contain a poisonous ingredient—from which the poison has been omitted. Their object is to escape the penalty incurred by infringing the Pharmacy Acts, and to that extent they are successful. The Pharmaceutical Society, however, is endeavouring to put a stop to the practice by taking proceedings under the Merchandise Marks Act, and there is some hope that in this way the offenders may be brought to book.

### THE EMPIRE HOSPITAL FOR PAYING PATIENTS.

For many months plans, announcements, and rumours have been under discussion for the construction of a new and up-to-date nursing home on a site in Vincent Square. These have now taken definite shape in the promotion of a limited company bearing the above title, with a capital of £25,000 in equal portions of 5 per cent. preference and of ordinary shares. From the particulars supplied in the prospectus, which has now been issued to the public, it appears that a new building is to be erected from designs by Mr. W. E. Hazell, especially for its intended purpose, and that it shall embody all the advances of hospital architecture. It is intended to take patients from three guineas a week upwards, and to supply them with the amenities of a first-class nursing home. The directors include a peer and a peeress (this step of including a representative of the sex is one whose wisdom in the circumstances cannot be doubted), a distinguished general, the two promoters of the company, one of whom is a medical man, and another gentleman. It is true that the medical representation on this board is (numerically) weak; but this defect is made up by an honorary advisory committee containing the names of some of the best-known consultants in London. In short, the new venture is one very much after the model of the well-known home at Fitzroy House, with this difference, that it is to be more of the nature of a commercial profit-making enterprise. If it is managed with the same regard for sound business principles, attains the same high standing of work and service, and commands to an equal degree the confidence of the medical profession, there is no reason why the Empire Hospital should not achieve the same measure of reputation and prosperity as its prototype in Fitzroy Square has so long enjoyed.

### THIS WEEK'S DRUG MARKET.

Business in the drug market has been depressed during the week owing to the disturbed state of the coal trade, which threatens to affect seriously the chemical and allied industries. So far, no advances in the prices of chemicals are traceable to the coal strike, but if the strike continues an advance in the price of a number of articles is almost inevitable. The anticipated dissolution of the Glycerine Convention has been avoided, the two important firms which had given notice of their withdrawal having signified their intention of remaining members of the Convention; under these circumstances the large reductions in the price of glycerine which would probably have taken place had the Convention been broken up are no longer expected; a smaller reduction in price, however, is expected to be announced. Cod-liver oil is substantially cheaper, and the price is still tending in a downward direction; favourable reports of the fishing continue to come from the Norwegian fishing grounds, and the indications are that the eventual total yield of cod-liver oil will be unusually large. In view of the downward tendency of prices it is still advisable to refrain from buying for the present. Senega root is dearer. Owing to the fact that buyers of opium have held aloof so long, there is a tendency, if only slight, towards lower prices for this drug, but it is likely that this tendency would disappear if any important purchases were made. Buchu leaves are dearer. Sugar of milk is still tending lower in price. Crude camphor shows signs of advancing in value, but there is at present no alteration in the price of the refined article. Cassia oil is dearer.

